# Supplementary material for: Fetal Exposure to Endocrine Disrupting-Bisphenol A (BPA) Alters Testicular Fatty Acid Metabolism in the Adult Offspring: Relevance to Sperm Maturation and Quality
Source: Int J Mol Sci. 2023 Feb 13;24(4):3769. doi: 10.3390/ijms24043769 (PMC9958878; doi:10.3390/ijms24043769)
Supplement: Supplementary file 1 [file ijms-24-03769-s001.zip › Supplementary Table S1.pdf]

Table S1: Predesigned SYBR green I rat primers and corresponding genes used for the mRNA expression analyses

Sl. no.	Primer ID	Gene symbol	Gene ID	Gene name	Nucleotide sequences (5'-3')	Ref_seqID
1	R1_Fabp9	FABP9	64822	Fatty acid-binding protein 9	F 5'- TAGCATTAGTTTCAATGGGG-3'<br>R 5'- GTTATAAGGCTCTTCACTTTCC-3'	NM_022854
2	R1_Cox2	COX2	29527	Cyclooxygenase 2	F 5'- CTCATACTGATAGGAGAGACG-3'<br>R 5'- TCGAACTTGAGTTTGAAGTG-3'	NM_017232
3	R1_Acsbg2	ACSBG2	301120	Long chain fatty acid-CoA ligase	F 5'- CATGACAACATCACATGGAC-3'<br>R 5'- TTTGATAGGGATCCAGATGTC-3'	NM_001080096
4	R1_Lpl	LPL	24539	Lipoprotein lipase	F 5'- CCTACTCCTTCTTGATTTACAC-3'<br>R 5'- GAAGATGACCTTTTTCTGAGTC-3'	NM_012598.2
5	R1_Lipe	LIPE	25330	Lipase E, hormone sensitive type	F 5'- GTGGAAAGATGTCAGGATATG-3'<br>R 5'- GTAAATCCATGCTGTGTGAG-3'	NM_012859
6	R1_Slc25a20	SLC25A20	117035	Carnitine-acylcarnitine translocase	F 5'- AGCCCACCTGTTATCCACTG-3'<br>R 5'- TGTGCAAAAAGAGCCTTCCT-3'	NM_053965.2
7	R1_Catsper2	Catsper2	366174	Cation channel sperm-associated protein 2	F 5'- TGTTGCTTGGTTCCATTATC-3'<br>R 5'- TTGACTGGTTCCTCTTAGTG -3'	NM_001012220
8	R1_Catsper1	Catsper1	689349	Cation channel sperm-associated protein 1	F 5'- AAACCCATCACCACTATGAG-3'<br>R 5'- CCAGATCTTTCCTGGTTTTG-3'	XM_001070492
9	R1_Fads1	FADS1	84575	Fatty acid destaurase 1	F 5'- GTACTTCTTCTTGATTGGAC-3'<br>R 5'- GTAAGTGAAGAAGACACGAAC-3'	NM_053445.2
10	R1_Fads2	FADS2	83512	Fatty acid desaturase 2	F 5'- CTTCTTCAATGACTGGTTCAG-3'<br>R 5'- CTTCAGTGAACTCACAATGTC-3'	NM_031344.2
11	R1_Elovl2	ELOVL2	498728	Fatty acid elongase 2	F 5'- CTTGTGGTCAAAGCTTCTTC-3'<br>R 5'- GAGGTATTTCTTCCACCAAAG-3'	NM_001109118.1
12	R1_Elovl5	ELOVL5	171400	Fatty acid elongase 5	F 5'- TTCTTCGTAAGAACAACCAC-3'<br>R 5'- ATAGTACGAGTACATGAGGAC-3'	NM_134382.2
13	R1_Degs1	DEGS1	58970	Delta 4 desaturase, sphingolipid 1	F 5'- ATCTTAGCGAAGTATCCAGAG-3'<br>R 5'- CAGAGTCATGGAATGGTTAAG-3'	NM_053323.2
14	R1_Scd-2	SCD 2	83792	Stearoyl-Coenzyme A desaturase 2	F 5'- TCCAGAGGAGGTATTACAAG-3'<br>R 5'- CTGTTTACAAACGTCTCACC-3'	NM_031841.2
15	R1_Scd-1	SCD 1	246074	Stearoyl-Coenzyme A desturase 1	F 5'- ATGAGAGAAGATATCCACGAC-3'<br>R 5'- AGTAAAATATCCCCCAGAGC-3'	NM_139192.2
16	R1_Fasn	FASN	50671	Fatty acid synthase	F 5'- AAAAGGAAAGTAGAGTGTGC-3'<br>R 5'- GACACATTCTGTTCACTACAG-3'	NM_017332.2
17	R1_Igf1	IGF1	24482	Insulin like growth factor 1	F 5'- GCACCTCCAATAAAGATACAC-3'<br>R 5'- TGGGCTTGTTGAAGTAAAAG-3'	NM_001082479
18	R1_Lep	LEP	25608	Leptin	F 5'- CTCATCAAGACCATTGTCAC-3'<br>R 5'- TGAGGATCTGTTGATAGACTG-3'	NM_013076.3
19	R1_Adipoq	ADIPOQ	246253	Adiponectin	F 5'- TGGCGATTTTCTCTTCATTC-3'<br>R 5'- AGGATTAAGAGGAACAGGAG-3'	NM_144744.3
20	R1_Act-B	ACT β	81822	Actin-beta	F 5'- AAGACCTCTATGCCAACAC-3'<br>R 5'- TGATCTTCATGGTGCTAGG-3'	NM_031144.3
