# Supplementary material for: Fetal Exposure to Endocrine Disrupting-Bisphenol A (BPA) Alters Testicular Fatty Acid Metabolism in the Adult Offspring: Relevance to Sperm Maturation and Quality
Source: Int J Mol Sci. 2023 Feb 13;24(4):3769. doi: 10.3390/ijms24043769 (PMC9958878; doi:10.3390/ijms24043769)
Supplement: Supplementary file 1 [file ijms-24-03769-s001.zip › Supplementary Table S2.pdf]

Table S2: The primary antibodies and their dilution used in this study

Target protein	Protein name	Primary antibody	Clonality	Host	Catalog & supplier	Dilution
FADS1	Fatty acid desaturase 1	Anti-FADS1	Monoclonal	Rabbit	#A0178 Abclonal	1:2000
ELOVL2	Fatty acid elongase 2	Anti-ELOVL2	polyclonal	Rabbit	#A17712 Abclonal	1:500
FABP3	Fatty acid binding protein 3	Anti-FABP3	Polyclonal	Rabbit	#AG25A0040 Adipogen	1:4000
FABP4	Fatty acid binding protein 4	Anti-FABP4	Polyclonal	Rabbit	#PA5-30591 Thermo	1:2500
FABP5	Fatty acid binding protein 5	Anti-FABP5	polyclonal	Rabbit	#A6373 Abclonal	1:1000
FABP7	Fatty acid binding protein 7	Anti-FABP7	Polyclonal	Rabbit	#A11604 Abclonal	1:1000
ADRP	Adipocyte differentiation related protein	Anti-ADRP	Polyclonal	Rabbit	#PA1-16971 Thermo	1:3000
PPAR α	Peroxisome proliferator activated receptor alpha	Anti-PPAR α	Monoclonal	Mouse	#MA1-822 Thermo	1:1000
PPAR γ	Peroxisome proliferator activated receptor gamma	Anti-PPAR γ	Monoclonal	Mouse	#SC-7273 Santacruz	1:500
Actin	Beta actin	Anti-β Actin	Monoclonal	Mouse	#A5316 Sigma	1:10000
